# Designs on consciousness: literature and predictive processing

**DOI:** 10.1098/rstb.2022.0423

**Published:** 2024-01-29

**Authors:** Karin Kukkonen

**Affiliations:** University of Oslo, Oslo, 0315, Norway

**Keywords:** literature, consciousness, predictive processing, embodied cognition

## Abstract

Predictive processing is a recent approach in cognitive science that describes the brain as an engine of probabilistic hierarchical inference. Initially proposed as a general theory of brain function, predictive processing has recently been expanding to account for questions of consciousness in philosophy and neuroscience. In my previous work (Kukkonen 2020 *Probability designs: literature and predictive processing*. New York, NY: Oxford University Press), I have shown how predictive processing can also be used to model our engagement with literary texts. In this article, I use my account of our engagement with literature in predictive processing terms, as well as recent work on predictive processing and consciousness, to explore how literature can shed light on various aspects of conscious experience, including qualia, counterfactual depth in conscious experience and sense of self. In the final section, I propose a number of theoretical questions that could be addressed by drawing on literature as a source of hypotheses and stimuli for possible experimental designs. The upshot is a picture where literature is not just a source of illustrative examples about conscious experience, but a designer environment through which we can explore and rethink consciousness.

This article is part of the theme issue ‘Art, aesthetics and predictive processing: theoretical and empirical perspectives’.

## 1. Introduction

When philosophers or psychologists discuss consciousness, they like to include quotations from literature: Tolstoy's Anna Karenina, Kafka's K and Woolf's Mrs Dalloway are said to provide a way into this elusive phenomenon. A long line of literary scholars have highlighted the literary devices for presenting consciousness [1], illustrated their role in the development of literary narrative [2], and outlined the features of ‘fictional minds’ [3]. Such encounters between the study of consciousness and the study of literature are by no means coincidental, since, as I am going to argue in this paper, literary narratives are a prime mode through which we as humans can explore consciousness.

In order to make this argument, I suggest we look at both literature and consciousness through the lenses of ‘predictive processing’, a recent approach in cognitive science that describes the brain as an engine of probabilistic hierarchical inference. Initially proposed as a general theory of brain function, predictive processing has recently been expanding to account for questions of consciousness in philosophy [4–6] and neuroscience [7,8], with a special issue on the topic in the journal *Review of Philosophy and Psychology* in 2022. Importantly for our ends, as I have shown in previous works (see especially [9,10]), predictive processing can also be used to model our engagement with literary texts. The notion of probabilistic hierarchical inferences seems to capture the way readers try to predict the verbal chain at different levels of abstraction, from sentences to characters' choices and mental lives, to plot developments and what is likely to happen in a given genre. Literary texts, for their part, seem to provide a designed sensory flow that is carefully crafted to sustain and engage readers’ probabilistic inferences. They are ‘probability designs’: artefacts designed to ignite our inferential capacities with unexpected events (prediction errors), formulations that make the perspective from which the narrative is told salient (precision and attention), as well as signals for genres (such as ‘crime fiction’ or ‘the modernist novel’) that best allow readers to make overall predictions (precision expectations) (see [9] for a full account of the model).

In this article, I use this description of our engagement with literature in predictive processing terms, as well as recent literature on predictive processing and consciousness, to explore how literature can shed light on various aspects of conscious experience, including qualia (§§2 and 3), counterfactual depth in conscious experience (§4) and sense of self (§5). In the final section (§6), I propose a number of theoretical questions that could be addressed by drawing on literature not just as a source of illustrative examples of conscious experience but as a source of hypotheses and stimuli for possible experimental designs. If my argument holds that the particular inferences enabled by probability designs in literary texts also allow readers to ‘watch themselves think’ [9, p. 191], then literature opens new possibilities for studying consciousness.

## 2. Strange inversions in consciousness

Do I experience something as warm simply because my perceptual apparatus responds to a source of heat? What about the particular subjective dimension of our experience of different kinds of warmth? Drawing on earlier work by Daniel Dennett, Andy Clark [4,5] proposes to think of these qualitative aspects of subjective experience (or ‘qualia’ in philosophical jargon) in terms of a ‘strange inversion’. ‘Strange inversions’, says Clark, ‘occur when things work in ways that turn received wisdom upside down’ [4. p. 202]. In the case of qualia (as in the case of causality for Hume), the strange inversion consists in the fact that ‘[w]hat appears to be a property of the world itself here turns out […] to be a property of the observer’ [4, p. 206]. In other words, an object would not make us feel and act in certain ways because it is warm (or red, or sweet, etc.), but it would be inferred to be warm (or red, or sweet, etc.) because it makes us feel and act in certain ways. Clark claims that we can understand this strange inversion in terms of Bayesian inference.

In a first step, Clark links conscious content to the general predictive processing model and defines it as based on ‘precision-driven best estimates of organism-salient (potentially action-driving) states of affairs' [5, p. 661, FN 43]. According to his picture, two ‘cycles’ of prediction run parallelly across multiple levels, namely, an outward-looking cycle of prediction targeting environmental stimulations and an inward-looking cycle that ‘targets our own changing physiological states—states involving the guts, viscera, blood pressure, heart rate and the whole inner economy underlying hunger, thirst and other bodily needs and appetites' [5, pp. 649-650]. These two cycles inform one another. Feeling warmth means at the same time detecting a change in our bodily states (interoception) and relating this change to a distal cause in the world (exteroception). The hypotheses developed in these cycles indicate ‘*what* I am seeing’ in exteroception and ‘*how* I am responding to that seeing’ in interoception [4, p. 215]. Combining them gives us the feeling of warmth that we consciously experience.

Literature captures such experience and puts it into words. In the Homeric epic, for example, the conscious experience of the sun on a given day is embodied in terms of the goddess Eos' ‘rose fingers’. In the modern novel, a character like Cora Sandel's Alberte can ‘envelop[…] herself in the heat like in a piece of cloth you can touch and feel’ [11, p. 30]. ‘What I am seeing’, such as rays of the sun, and ‘how I am responding to that seeing’, such as perceiving delicate touches of warmth, are entwined in the verbal phrase of Homer's ‘rose fingers’ and in Sandel's evocative passage.

So what is this feeling of warmth that we consciously experience, after all? Clark addresses the example of ‘sweetness’, ‘cuteness’ and colour, and argues that these qualia are not produced by properties of the stimulus, but rather by ‘the subtle complex of reactive dispositions' of the perceiving subject [5, p. 652]. So, to continue with our warmth quale from above: such quale arise from a number of predispositions and memories of previous experiences, such as the softness of rose petals and the sensation of putting on a warm cloak. ‘The complex of reactive dispositions comes first, and we label things which evoke that complex of responses as ‘sweet’ [or ‘warm’]’ [5, p. 653]. However, we are not normally aware that reactive dispositions come first and qualia follow, and therefore project the quality of ‘delicate warmth’ from ourselves on the world. The linguistic term we use to indicate these qualities then becomes a ‘shorthand’ or, rather, a conceptual short-cut that gets ‘deployed by the system itself as a means of better predicting its own and others' behaviours’ [5, p. 653].

In a predictive processing perspective, then, feeling the qualitative aspects of experience, or qualia, is not different from inferring the presence of objects in our environment starting from the stimulations hitting our sensory organs. ‘Just as the PP agent infers the existence of dogs, cats, tables and chairs (as a neat way of making predictive sense of the sensory flux), so too she infers the presence of rather puzzling ‘qualitative features' such as the cuteness, brownness, or annoyingness of the dog’ [5, p. 659]. If we find qualia more puzzling, says Clark, this is just ‘due to the extent to which they involve greater degrees of hidden reference to our own inner physiological states and reactive dispositions' [5, p. 659, FN 39]. Clark links qualia especially to ‘the ability of advanced cognizers to model themselves and their own reactive dispositions’ [5, p. 662]. It is because of this ability to model our own reactive dispositions, arguably linked to language and other cultural means of scaffolding experience and making it retrievable, that our knowledge of the world and our readiness to connect our bodily states with hypotheses about the external world can get integrated in the conscious experience and the sense of ‘puzzlement’ that accompanies it [5, p. 662]. It is therefore the response in the perceiver that leads to the experience of pleasant warmth, and in the case of qualia, we somehow know it.

The puzzlement that surrounds the problem of qualia may also derive, as Clark *et al*. [12] suggest, from a disconnect between our higher-level understanding of the world and our experience of it in qualitative terms. Qualia feel highly certain because they depend on well-established, mid-level inferences about the shape of our world. Remember that from a predictive processing perspective to perceive is to make inferences about the causes of our sensory inputs at different levels of abstractions. Now some mid-level inferences might feel subjectively certain despite lending themselves to many alternative higher-level explanations. In the example of Clark *et al*., we perceive a fountain and assume that the liquid emerging from it is water and not vodka, even though we have not actually tested the counterfactual possibility of an alcoholic fountain. ‘But as the depth and reach of our generative models increased, we became aware that things are not always as they seem’ [12, p. 24]. Despite this higher-level doubt, however, agents like us ‘remain forced to experience the world *as if* these states were known with total certainty’ [12, p. 25]. 'They are then led to represent some of their representations (the highly certain mid-level encodings) as deeply special, opening the door to all the demons of the Cartesian mindset’ [12, p. 32]—and also, I would add, to some of the most interesting effects of literature.

## 3. Strange inversions in literature

Clark's suggestions about consciousness in the predictive processing framework do not make explicit much in the way of encultured cognition—i.e. cognition shaped by cultural technologies like language, reading and writing or through the scaffolding of human cultural designer environments [13,14]. However, he does mention linguistic terms as a possibility for creating shorthands in the inferential processes that determine the subjective dimension of conscious experience [4, p. 653]. Language, reading and writing clearly offer key extensions to the process sketched in Clark's series of articles. The phrases from Homer and Sandel work themselves as ‘linguistic shorthands', but they are also something that readers can point to in the text, identifying their own ‘reactive dispositions’, partly because the shorthand appears stabilized in the written text, partly because the literary language is ‘strange’ and draws attention to itself. Even the most canonical or most realist literary work can be ‘strange’ in this respect: Homer likens the rays of the sun to a (giant) goddess' fingers reaching through the clouds, and Sandel describes ambient warmth metaphorically as a piece of cloth. Indeed, I would argue that literature, which builds on the cultural technologies of language, reading and writing, and which develops a special designer environment for human thought, generally opens new possibilities for shaping the awareness of our conscious states, rather than merely representing them through language and narrative.

It has been claimed that literary texts do not feature representations of consciousness at all. ‘Alberte envelops herself in the heat like in a piece of cloth you can touch and feel’, relates conscious experience only when readers, first, assign that conscious experience not to themselves but to a character (consciousness attribution) and, second, run an embodied simulation of the language (consciousness enactment; see [15] for this distinction). I would agree but add that in literary texts this is rarely a seamless process. The designed sensory flow of language in a literary text, its probability design, scaffolds thought processes in such a way that makes consciousness more graspable—modelling it without representing it.

How can we think about this capacity of literature? Language as a system of ‘material symbols’ [16] complements other cognitive processes: it can redirect attention, leading to priming effects in perception [17]. It can also be used in the cognitive control of skilled action, such as in the case of ‘prompts’ that realign and adjust movement patterns [18]. Also, finally, in its written form, language becomes available to manipulation that extends thought [19]. I propose that the argument also applies to the literary text. Clark and Lupyan speak about language in general, Sutton *et al*. address individual spoken phrases, while Menary speaks about writing in general. The effect of language and writing does not necessarily lead to an enhanced understanding of conscious experience in any of these cases. However, the way in which literary probability designs manipulate language and writing arguably increases the potential of material symbols to bring consciousness into focus.

Another way literary texts bring consciousness into focus is by promoting an inversion in the normal balance of exteroception and interoception. As we have seen through the microexamples from Homer and Sandel, literary texts tend to integrate expressions referring to interoceptive and exteroceptive sensations on a linguistic level. Across longer sequences of text, such an integration can achieve the particular effect of ‘presence’, that is, the feeling of immersion in fictional worlds [20]. Such an immersion in fictional worlds is often facilitated by the material setting in which reading takes place, a setting that usually differs significantly from our everyday world. Engaged readers focus on the rather ‘poor’ stimulus of letters on a page and tend to ignore the perceptually rich material environment in which they are physically located. The literary text is ‘stable’, and readers generally prefer a stationary position with as little distraction as possible when they engage with a book. Normally, we aim to keep interoception stable, so that we can explore our world and make new exteroceptive explorations [21,22]. In engaging with literary texts, however, we tend to keep exteroception (largely) stable, so that we can explore our inner worlds, in a space that ranges from immediate physical responses to larger models of the conscious self (see [23]). Reading literature, therefore can be seen as a cultural technique for bringing interoception to the fore, similar to mindfulness meditation. To my knowledge, no empirical studies have addressed this phenomenon yet, but in an article arguing for literature as a special mode of access to interoception, I have made a number of proposals for how this could be investigated [23]. In that sense, literary texts enable readers to ‘materialis[e] and confront […] [their] own high-level assumptions' [6].^1^ Miller *et al*. privilege other examples, such as psychedelic drugs, but these arguably cannot match literature when it comes to the sustained and stable entwinement of immersive distance from the world and precision in reflection. This inversion in the balance between exteroception and interoception, based on the ‘strangeness’ of the reading situation, is the decisive stepping stone towards thinking of literature as a cultural technology for consciousness exploration in the predictive processing framework.

Moreover, literary texts encourage an exploration of our conscious states that is both immersive and reflexive by providing readers with what I would call ‘consciousness proxies', namely, reference points through which they can model their own inferences. Literary characters serve this purpose: when Alberte in Sandel's novel shudders from the cold in the north-Norwegian coastal town, to some extent, readers feel a resonance of this embodied experience. Moreover, literary texts then also shape the background against which this reference point is set: they build, as it were, their own situation models (see also [24, p. 266] who refers to this as ‘Umwelt construction’). Sandel's narrator, for example, usually adopts a focalization on (that is, the embodied perspective of) Alberte, but it is also clear that Alberte does not narrate her experiences herself. Indeed, it has been pointed out that Sandel resorts to irony to create moments of distance from her character's experience (see [25]). The entire Alberte trilogy is narrated in the present tense. This is an unusual choice of narrative fiction, which usually depends on a temporal distance and dynamics between the time at which the story plays out in the fictional world (narrated time) and the time it takes to tell the story (time of narration; see [26] for the classic formulation of this distinction). Sandel achieves an effect of repetition and iteration through narrating the same event (apparently) multiple times and through deploying the present tense [27]. These analytical points from literary studies provide a glimpse of how Sandel reshapes experience through the design of the sensory flow of her language as it unfolds, line by line and page by page. The reshaping entwines ‘consciousness proxies' and situation models, for example, when Alberte's boredom is reinforced by the constant present and iterative nature of narrative time.

Literary probability designs, then, provide a scaffolding for readers' inferential activities that: (i) offer stable ‘linguistic short-hands’ for qualia that register as particularly salient owing to the strangeness of literary language and to which readers can return, reading after reading, for further reflection; (ii) limit the pressure of external circumstances; and (iii) enable a distancing effect through ‘consciousness proxies' and the construction of unfamiliar situation models. By these different means, a literary probability design makes the potential for ‘strangeness’ in qualia and conscious experience especially salient. What is more, given that the engagement of a literary text normally unfolds across long temporal extensions (for example when reading a novel across multiple sittings), literary texts offer a sustained process of engaging with one's consciousness.

## 4. Counterfactual depth and multiple temporalities

The fact that our engagement with literary texts normally occupies a considerable temporal extension also maps onto accounts of consciousness in predictive processing that stress the importance of temporal thickness and counterfactual depth. Karl Friston for example makes the conceptual step from self-organizing systems in the predictive processing framework to self-conscious agents by positing that ‘the mind comes into being when self-evidencing has a temporal thickness or counterfactual depth, which grounds inferences about the consequences of *my* actions' [8]. Counterfactual depth in experience refers to a sense of having access to multiple different possibilities of interaction with the world. For example, the imagined consequences of our bodily movements through the world might lead us to develop a rich experience of being in the world and, thereby, the basis for our sense of self [28].

Literary texts, like Sandel's novel, present readers with Alberte's perception of the world around her:

Alberte envelops herself in the heat like in a piece of cloth you can touch and feel. She is never too warm. She feels her hands differently and her body and her face, she quickly sews the tablecloth for Mum and does not really know whether she thinks this is entertaining or whether it is just the warmth and the bright light that makes it feel thus. (my translation; original: Alberte svøper sig i heten som i et stof, man kan ta og føle på. Hun får det aldrig for varmt. Hun kjender sine hænder andnerledes, og sin krop og sit ansikt, syr fort på bordløperen til mamma og vet ikke riktig selv, om hun synes det et morsomt eller om det bare er varmen og det lune lyset, som får det til at kjendes så.) [11, p. 30].

Conscious perceptual experience, as a rule, comes with a sense of various possibilities of interacting with the world. Literature offers enhanced experiences of this kind. Consider Sandel's use of Alberte as a ‘consciousness proxy’ here: The exteroceptive cycle of perceiving the heat ‘like a piece of cloth’ depends exactly on an imagined interaction potential or sensorimotor contingency of how one could ‘touch and feel’ and drape said piece of cloth. Sandel immediately integrates it with the interoceptive cycle and extends this dimension in the next sentence that foregrounds how Alberte feels her hands, body and face. At the same time, also Alberte's sense of self emerges from the tension between feeling so good and the boring and tedious task of sewing a tablecloth for her mother with whom she has a rather difficult relationship. She would not expect to feel good while performing this activity and wonders whether there is an entertaining aspect in the activity itself or whether it is the setting in the warmth and light of the well-heated living room, sitting together with her friends, that generates this experience in her. Readers may indeed experience the ‘puzzlement’ typical of qualia thanks to the strangeness of literary language. Note that while Alberte enacts the metaphorical link between cloth and heat, the text does not explicitly liken the ‘cloth’ of warmth to the table cloth (indeed, these are two different words in the Norwegian: ‘stof’ and ‘bordløper’). That link ought to be made by the reader who also needs to contextualize the tension between expectations about physical and mental comfort that Alberte experiences, for example, by paying closer attention to the role of warmth when reading scenes featuring Alberte. Alberte's readers, in other words, resemble the puzzled perceivers in Clark, Friston and Wilkinson's example [12], because the certain experience that ‘she feels her hands differently’ gives rise to a whole range of possible explanations. Alberte herself ponders some of these explicitly, while readers may infer others. However, while puzzlement in an everyday context soon evaporates as perceivers go about their activities, the puzzlement of Alberte's readers can linger.

We can see immediately also the multiple timescales at play in this counterfactually rich moment: (i) right now, Albert feels warm; (ii) but usually or ‘never’ does she feel like this (and this information takes readers into the deep dimension of her personal memory, which the novel, overall, reinforces through it repetitive use of tense and narrative structures); and (iii) and she does not know whether in a future reiteration of the sewing exercise her feeling would be repeated, because it may well just be owing to the need for warmth that is satisfied in this particular instance. Here, I only discuss a short passage. Given, however, that this is embedded into a larger stream of narrative, individual elements (such as the sewing project with the tablecloth, and other instances where Alberte warms up after a chilling walk in the winter or through encounters with others) also link to multiple temporalities that run across the entire sequence of the text (see [29] for an account of narrative's multi-temporal structures in reading). The different counterfactual perspectives can be mapped onto the different temporalities that unfold in parallel in the narrative. The embodied experience of readers is arguably deepened across multiple levels of linguistic meaning-making, but the counterfactual depth of experience is also given a particular focus and stability in the linguistic form, which anchors readers' reflection on how what they read might relate to their own experience [23,30].

In this miniature scene, Sandel manages to compress the experience of Alberte, torn between the desire for physical and interpersonal warmth and her struggle with the domestic expectations of her family. The tablecloth remains inert in contrast to the dynamic experience of the heat that ‘envelops’ Alberte, representing the repressive aspects of domestic activities. Complex interpersonal and psychological constellations are encapsulated in a focused embodied image. A handful of sentences from Sandel maps out an entire world of embodied, enculturated experience: immediate interoception is linked with physical positions in the world and the perception of this world, and then on to larger interpersonal relationships and the individual's place in society. As a result, the passage provides a ‘shorthand’ for a complex, subjective experience endowed with a counterfactual depth and temporal thickness that mirror those underlying consciousness. The perceivers of the alcoholic fountain usually do not have the occasion or leisure to prove their perception by exploring alternatives, while readers, supported by the counterfactually rich and temporally deep structures of a literary texts can spend time on assessing counterfactual alternatives (bounded by ‘consciousness proxies') and thereby roam the space between high-level doubt and mid-level certainty underlying qualia.

## 5. Selves, consciousness and reading literature

Two intertwined lines of argument about the self and consciousness can be distinguished in the predictive processing literature. First, selves exist only insofar as they keep revisiting a limited number of predictable states. As Karl Friston puts it: ‘You are you because you revisit (the neighbourhood of) these attracting states time after time’ [8, p. 3]. Second, the conscious experience of being a self depends on temporally thick and counterfactually deep modes of inference [7,8,28]. These inferences may remain below the threshold of consciousness. The next step in my argument is to show how literary texts may both provide the focal points for ‘consciousness proxies' and larger reaches of pre-conscious experience that is temporally deep and counterfactually rich.

Literary texts in particular build up the temporal thickness and counterfactual depth that Friston and Seth see as a precondition for the experience of being a conscious self in their accounts. As we saw, counterfactual depth is generated by the multiple perspectives encoded in literary texts, including, for example, the third-person narrator in Sandel who narrates Alberte's personal experience, and the entwinement of exteroceptive and interoceptive embodied language in literary texts. What about temporal thickness? With its 250 pages, a novel like Sandel's *Alberte and Jakob* [11] extends at least over six or seven hours of readers' lived experience. If readers choose not to complete the novel in one sitting, it may extend over several days and perhaps even weeks. Across the multiple levels of their probability designs, literary texts enable such a temporally extended engagement: the overall shape of the plot creates consistency in the narrative (on the first-order probability design), the modulating of the situation model through sequences of focalization, sometimes reflected in explicit chapter divisions, paces readers’ patterns of attention (on the second-order probability design), and the precision expectations based on a text's genre or intertextual references creates a further layer of patterned coherence (on the third-order probability design). Readers can easily leave a literary text for a couple of hours or a couple of days and return to the next page, picking up the threads of plot, patterns of attention etc. without too much disorientation, arguably because the text meshes productively with readers’ own temporally thick consciousness. This capacity of the probability design distinguishes literary reading from other activities, such as dreaming or mind-wandering, which appear similarly detached from the sensory flow coming from the real world. In literary texts, a consistent experience that extends over long stretches of time is enabled.

Reading literature, I have argued, is marked by multiple temporalities that unfold all at the same time. The speed of reading is usually measured at the rate of words read per minute. However, readers grasp the meaning of a text at different levels, ranging from the recognition of individual words, their phonetic and semantic content, to the way in which they are embedded in larger paragraphs ([29], drawing on [31]) and narrative structures. In other words, literary probability designs create ways to align different lines of inferencing subjective time. Some meaning-making processes are very swift and automatic, while others unfold across larger temporal frames and can be explicitly addressed by narrators or characters. Indeed, the articulation of characters' temporal experience can serve readers as what I have called a ‘consciousness proxy’ through which they run their own generative models, either in alignment with their own experience of time or in contrast to it.

Literary texts enable long temporally extended engagements that range over multiple levels of meaning-making. They are exceptional tools for generating a temporally thick experience, both in the representation of fictional minds as consciousness proxies and in establishing self-relevance for the readers' own minds. Linguistic structures can elicit mind-wandering in readers (for example, remembering related events from their own lives and also imagining how they would have reacted in a similar situation; [30]), but they also provide structures of attention that allow readers to find their way back into the probability design and continue reading [32], now enriched by the contrast or parallel to their own selves. Friston associates the self with action selection among counterfactuals, and more specifically, with the temporal orientation of action selection: ‘the sort of selection that we have associated with (self)consciousness operates within the same system—a system that can simulate multiple futures, under different actions, and select the action that has the least surprising outcome’ [8, p. 5]. In reading literature, readers can simulate multiple futures unfolding across different levels of temporal extension and relate them to their own experience, and what is more, they can also entertain the most surprising or the strangest outcome. Literary texts have the potential to redirect and refocus readers' attention to make sense of their own temporally deep, self-conscious inferences; these literary experiences may then be recorded in personal memory along with autobiographical memories [33], providing a further room of resonance in the counterfactual construct of the self according to predictive processing.

## 6. Strangeness, ‘found science’ and experimental designs on consciousness

Empirical work within the predictive processing framework has strongly privileged visual art and music (see [34,35]). Koelsch *et al*. argue that ‘Music offers a powerful tool to investigate PC [ = predictive processing] in the brain, because the statistical regularities in music are so well defined’ [35, p. 74]. Such an argument could not be made for literature. However, literary texts show the same possibilities for aesthetic emotions (facilitated through motor inhibition, see [34]) and (epistemic) active inference (see [35]) that are said to underlie aesthetic pleasure.^2^ In this article, I aimed to show how literary texts rework conscious experience and make the dispositions underlying it graspable. As such, they have the potential to provide hypotheses for studying consciousness in empirical and theoretical contexts, as a kind of ‘found science’ where the experimental set-up is developed through an investigation of the historical practice of authors. The approach has been proposed by Cavanagh [36] for the visual arts and can arguably also be applied to the literature [37]. Moreover, I argue, integrating insights from the literature into experimental design may also expand the possibilities of empirical work on consciousness. What then are the elements of consciousness on which literature can provide new perspectives?

(i) An experimental setting in which the balance point between interoception and exteroception is shifted. I have argued that literary texts stabilize exteroception in the reading process, so as to enable more adventurous exploration in interoception. Studying bodily markers of interoception, such as the awareness of heart-rate, breathing rate or skin-conductance, on readers engaged in literary texts may enable comparisons with everyday scenarios where the point of balance is located on the exteroceptive end [38].
(ii) A flexible take on the entwinement of conscious/controlled and pre-conscious/automatic processes in skilled action. Skilled action, such as reading, depends on automatic processes but also integrates conscious experience (see [18]). A comparative study of how readers of a written text and how listeners of an audiobook version respond to strange ‘linguistic short-hands' or ‘prompts’ for reflection, in terms of attention for example, could serve to study how and where conscious and pre-conscious aspects of skilled action are integrated around prompts and how much of this depends on the visual or auditory modality.
(iii) A better grip on the ‘puzzlement’ evoked by qualia. Clark [5] proposes that the puzzling nature of qualia depends on the fact that they are inferences made with higher precision than they should have. Literary texts need to construct their own situation models as the background against which they posit qualia. In order to generate theoretical and empirical hypotheses when designing experiments around qualia one could investigate how literary texts construct the background for the puzzlement of qualia from words across multiple counterfactual possibilities (for example in the minds of characters) and in pacing temporal depth (for example in the online construction of the fictional world).
(iv) Understanding the temporal dynamics of affect in conscious experience. The inferential work that unfolds over multiple temporal scales while reading a literary text arguably has an affective dimension. Here, readers' emotional investment in certain predictions, the narrative pacing of how these predictions become more or less probable, and the emotional valence linked to the reduction of prediction error [39] could be examined. An experiment, potentially featuring electroencephalography, eye-tracking and motion capture, could for example manipulate the levels of prediction errors in narrative (across multiple temporal scales; see [35] for the case of music) in order to study the evolving affective profile that accompanies conscious experience. A study along those lines could shed light on ‘affect [as] the foundational form of consciousness’, as Mark Solms proposes it ([40, p. 157]; italicised in the original). Readers' sense of agency in these processes (as scaffolded by literary texts) may then, finally, also reveal more about the dynamics of agency and passivity in surfing the stream of conscious experience.

Each of these proposals depends on the notion that literature does strange things with our thinking, all the way from pre-conscious ‘feelings’ to conscious and controlled experiences. These ‘strange things’, analysed by literary theory and conceptualized through models informed by predictive processing, arguably provide a treasure trove for the further theoretical and empirical study of consciousness along these lines.

## Data Availability

This article has no additional data.

## Endnotes

1 I would like to thank the reviewer who drew my attention to this piece in the revision stage.

2 See Kukkonen [9, pp. 76–77] for motor inhibition / sensory attenuation in literature, and pp. 54–55 for epistemic active inference.
